# Rhinovirus Attenuates Non-typeable *Hemophilus influenzae*-stimulated IL-8 Responses via TLR2-dependent Degradation of IRAK-1

**DOI:** 10.1371/journal.ppat.1002969

**Published:** 2012-10-04

**Authors:** Benjamin L. Unger, Andrea N. Faris, Shyamala Ganesan, Adam T. Comstock, Marc B. Hershenson, Umadevi S. Sajjan

**Affiliations:** 1 Department of Pediatrics and Communicable Diseases, University of Michigan, Ann Arbor, Michigan, United States of America; 2 Department of Molecular and Integrative Physiology, University of Michigan, Ann Arbor, Michigan, United States of America; McMaster University, Canada

## Abstract

Bacterial infections following rhinovirus (RV), a common cold virus, are well documented, but pathogenic mechanisms are poorly understood. We developed animal and cell culture models to examine the effects of RV on subsequent infection with non-typeable *Hemophilus influenzae* (NTHi). We focused on NTHI-induced neutrophil chemoattractants expression that is essential for bacterial clearance. Mice infected with RV1B were superinfected with NTHi and lung bacterial density, chemokines and neutrophil counts determined. Human bronchial epithelial cells (BEAS-2B) or mouse alveolar macrophages (MH-S) were infected with RV and challenged with NHTi, TLR2 or TLR5 agonists. Chemokine levels were measured by ELISA and expression of IRAK-1, a component of MyD88-dependent TLR signaling, assessed by immunoblotting. While sham-infected mice cleared all NTHi from the lungs, RV-infected mice showed bacteria up to 72 h post-infection. However, animals in RV/NTHi cleared bacteria by day 7. Delayed bacterial clearance in RV/NTHi animals was associated with suppressed chemokine levels and neutrophil recruitment. RV-infected BEAS-2B and MH-S cells showed attenuated chemokine production after challenge with either NTHi or TLR agonists. Attenuated chemokine responses were associated with IRAK-1 protein degradation. Inhibition of RV-induced IRAK-1 degradation restored NTHi-stimulated IL-8 expression. Knockdown of TLR2, but not other MyD88-dependent TLRs, also restored IRAK-1, suggesting that TLR2 is required for RV-induced IRAK-1 degradation.

In conclusion, we demonstrate for the first time that RV infection delays bacterial clearance *in vivo* and suppresses NTHi-stimulated chemokine responses via degradation of IRAK-1. Based on these observations, we speculate that modulation of TLR-dependent innate immune responses by RV may predispose the host to secondary bacterial infection, particularly in patients with underlying chronic respiratory disorders.

## Author Summary

Rhinovirus (RV) is responsible for the majority of common colds. RV infection is also associated with hospitalizations for lower respiratory tract illness, a significant proportion of which are accompanied by bacterial infections including acute otitis media, sinusitis and pneumonia. However, the mechanisms by which RV increases susceptibility to secondary bacterial infections are not understood. In this report, we demonstrate for the first time that RV infection promotes bacterial persistence of non-typeable *Hemophilus influenzae* (NTHi) *in vivo*, which was associated with reduced expression of neutrophil-attracting chemokines and neutrophil infiltration into the lungs. Further, RV infection attenuated NTHi or TLR2 or −5 agonist-stimulated chemokine responses in cultured bronchial epithelial cells and alveolar macrophages, suggesting that RV interferes with TLR-related innate immune responses. Next, we found that RV infection caused rapid degradation of IRAK-1, a key adaptor protein in the MyD88-dependent signaling. Inhibition of IRAK-1 degradation restored NTHi-stimulated chemokine responses in RV-infected bronchial epithelial cells. Finally, reductions in IRAK-1 were dependent on TLR2. Together, our results suggest that RV may increase the risk of acquiring secondary bacterial infection by attenuating TLR-dependent innate immune responses.

## Introduction

Respiratory infection by one pathogen can alter the innate immunity to unrelated pathogens long after resolution of the first infection. This can affect the pathogen clearance and increase disease severity [1]. Severe illness or death due to bacterial infection following viral influenza is one of the most well-documented instances of this phenomenon [2]. Secondary bacterial infections (otitis media, sinusitis, pneumonia) have also been demonstrated following infection with respiratory syncytial virus, enterovirus and rhinovirus (RV) [3], [4], [5]. In addition, RV triggers exacerbations of chronic obstructive pulmonary disease and cystic fibrosis, conditions in which the airways are chronically colonized with bacteria [6], [7].

Most studies examining this problem to date have been focused on understanding the molecular mechanisms by which influenza virus predisposes host to secondary bacterial infection. Influenza is a lytic virus which causes extensive damage to the airway epithelium, thereby increasing exposure of bacterial receptors and inducing apoptotic cell death of macrophages and neutrophils [8], [9], [10]. IFN-γ production during influenza infection decreases the phagocytic and anti-bacterial capacity of alveolar macrophages [11]. Immunosuppressive cytokines such as IL-10 and TGF-β produced after influenza virus infection have been proposed to modify the initial chemokine response to subsequent bacterial infections [12], [13]. Glucocorticoids induced by influenza virus suppress pro-inflammatory cytokine responses to secondary bacterial challenge [14]. In addition to immunosuppressive molecules, antiviral proteins (such as IFNs) produced during viral infections attenuate initial KC/CXCL-1 and MIP2/CXCL2 responses to secondary pneumococcal challenge, resulting in increased persistence of bacteria and death in mouse models of infection [15]. Influenza virus also desensitizes TLR receptors *in vivo* and decreases pro-inflammatory cytokine responses to bacterial ligands long after the viral infection resolves [16].

Unlike influenza virus, RV does not cause excessive cell damage. Yet, RV infection has been shown to precede otitis media and acute lower respiratory tract infections requiring hospitalization, each of which are associated with bacterial infection [3], [4], [5]. A handful of studies have demonstrated that RV infection enhances bacterial adherence by increasing the expression of host molecules that serves as receptors for bacteria, such as ICAM-1, platelet-activating factor receptor and carcinoembryonic antigen-related cell adhesion molecule [17], [18]. RV infection was also shown to promote internalization of *S. aureus* into non-fully permissive lung epithelial cells [19]. In addition, RV infection disrupts barrier function and promotes transmigration of bacteria across the polarized airway epithelium [20], [21]. RV was recently shown to attenuate cytokine responses to subsequent challenges with two bacterial products, LPS and lipoteichoic acid, in alveolar macrophages [22]. However, the consequences of such RV-induced chemokine suppression on subsequent bacterial infection have not been demonstrated *in vivo* or *in vitro*. Further, the mechanism by which RV infection suppresses cytokine responses to subsequent TLR stimulation has not been determined.

In the present study, we show for the first time that RV promotes persistence of non-typeable *Hemophilus influenzae* (NTHi) by suppressing neutrophil-attracting chemokine responses. We also demonstrate that RV suppresses NTHi-induced IL-8 expression in airway epithelial cells and alveolar macrophages by inducing TLR2-dependent degradation of IRAK-1.

## Results

### RV infection promotes NTHi persistence *in vivo* by suppressing chemokine expression and neutrophil recruitment

**Figure 1:**
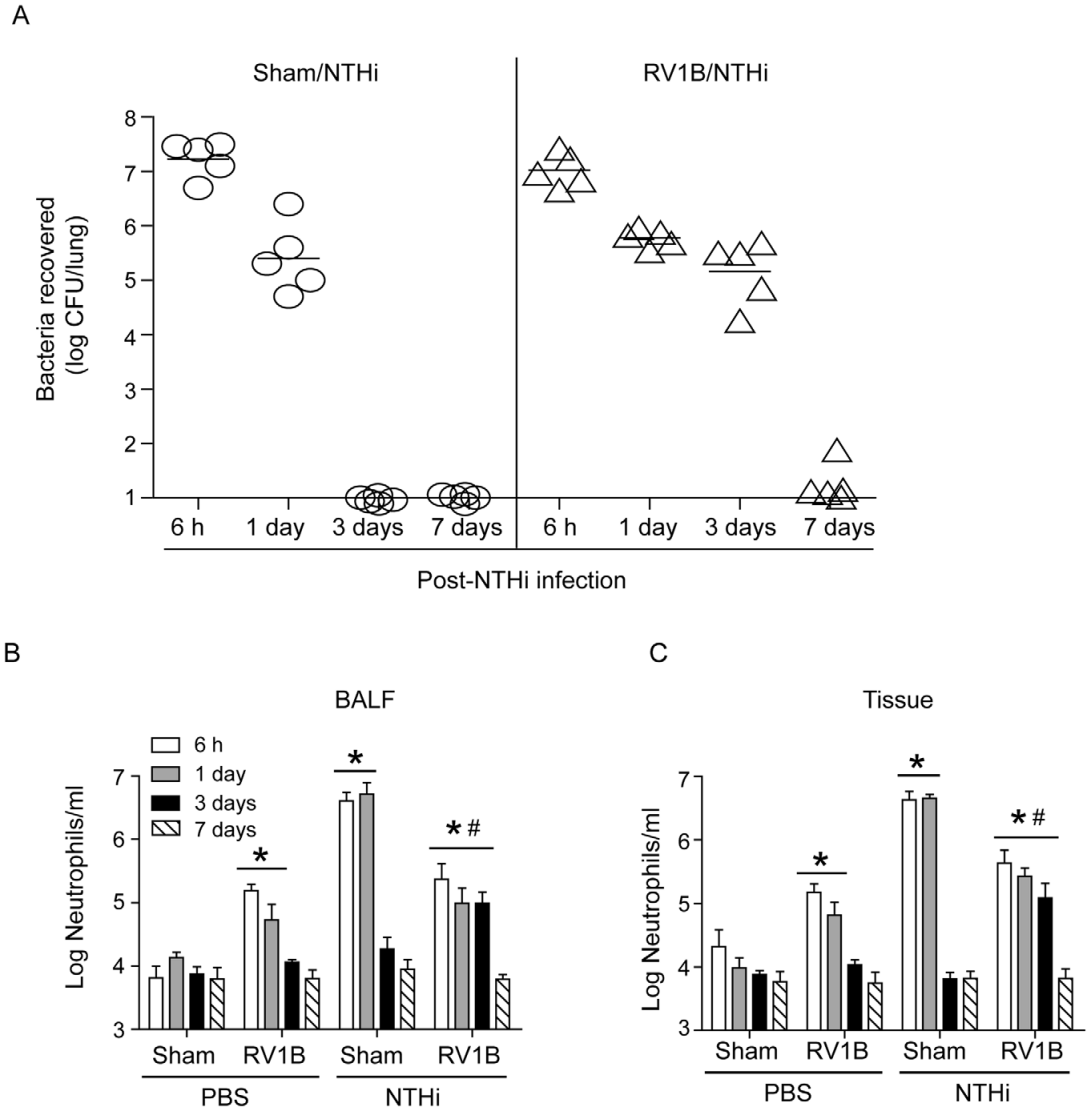
RV infection promotes bacterial persistence and decreases neutrophil infiltration to subsequent bacterial challenge *in vivo*. BALB/C mice were infected with RV1B or sham by intranasal route. Two days later, mice were infected with NTHi or treated with PBS via intratracheal route and sacrificed at 6 h, 1 day, 3 days or 7 days post-NTHi infection. (A) Mice were sacrificed and lungs collected aseptically, homogenized in sterile PBS and 10 fold serial dilutions of lung homogenates were plated to determine bacterial density. (B) Cytospins of BAL cells and (C) lung digests enriched for leukocytes were prepared, stained with Diffquick and number of neutrophils were counted. Data in A represent mean and range and in B and C, mean±SD (n = 4–9, * p≤0.05, two-way ANOVA, different from sham; # p≤0.05, two-way ANOVA, different from sham/NTHi infected animals).

Major group rhinovirus, such as RV39, which binds to ICAM1 does not infect murine cells due to species specific variations in the ICAM-1 D1 extracellular Ig domain [23]. Previously, we have demonstrated the feasibility of infecting mice with RV1B, a minor group virus, which binds to low-density lipoprotein family receptors [24]. Therefore, in these experiments we used minor group virus, RV1B. Mice were infected with sham or RV1B by the intranasal route and two days later superinfected with NTHi by the intratracheal route. Chemokine expression and bacterial load in the lung were assessed 6 h and 1, 3 and 7 days post-NTHi infection. Although, there was no difference in the lung bacterial load between sham/NTHi and RV1B/NTHi groups at 6 and 24 h post-NTHi infection (Figure 1A), RV1B/NTHi group showed significantly less airway and interstitial neutrophils than sham/NTHi group at these time points (Figure 1B and 1C). While mice in sham/NTHi group cleared all bacteria by 72 h post-infection, RV/NTHi-infected animals showed bacteria in their lungs at low levels which were associated with increased number of airway and interstitial neutrophils. By 7 days post-NTHi infection, RV/NTHi-infected animals cleared all bacteria from their lungs and showed neutrophils counts similar to uninfected animals. Compared to sham-infected mice, RV-, sham/NTHi- and RV/NTHi-infected animals showed significant increases in both airway and interstitial lymphocyte counts 3 and 7 days post-NTHi infection (Supplemental Figure S1A and S1B). However, there was no difference between RV, sham/NTHi and RV/NTHi groups. Only the RV/NTHi group showed a significant increase in the number of macrophages/monocytes 3 and 7 days post-NTHi infection compared to sham-infected mice (Supplemental Figure S1C and S1D).

**Figure 2:**
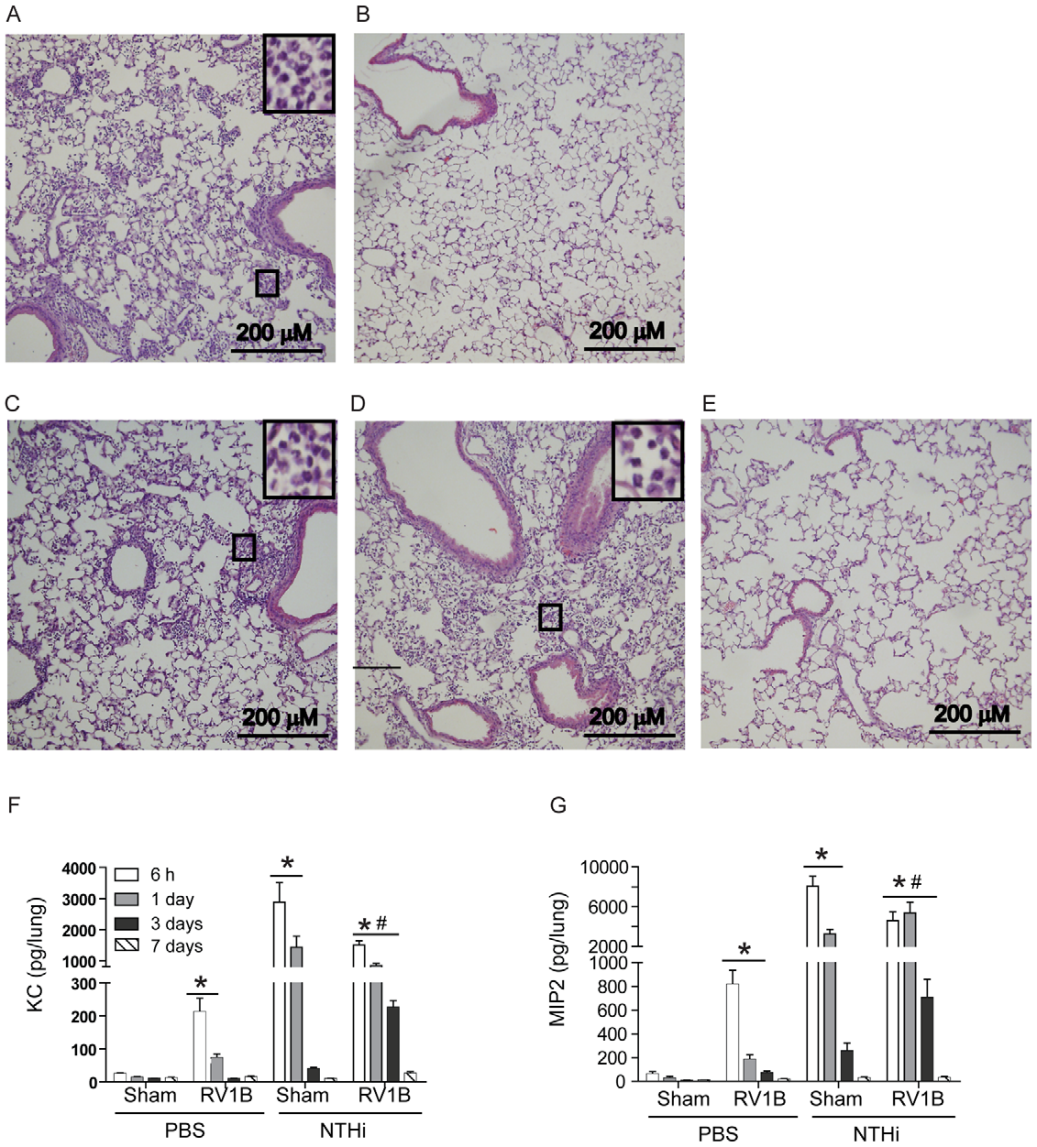
RV infection prolongs lung inflammation caused by NTHi and suppresses initial chemokine response. Mice were infected with sham or RV followed by NTHi as described in Figure 1. (A and B) Hematoxylin and eosin stained lung sections from Sham/NTHi-infected mice at 1 and 3 days post NTHi infection respectively. (C to E) Hematoxylin and eosin stained lung sections from RV1B/NTHi-infected mice at 1, 3 and 7 days post NTHi infection respectively. Inserts in the top left corners of A, C and D are magnified view of area marked in respective panels showing neutrophils. (Fand G) Chemokine levels in lung homogenate supernatants were determined by ELISA. Images in A to E are representative of 3 to 4 animals per group. Data in F and G represent mean±SD (n = 4–9, * p≤0.05, two-way ANOVA, different from sham; # p≤0.05, two-way f ANOVA, different from sham/NTHi infected animals).

Histologic evaluation of lung sections, revealed diffuse neutrophilic inflammation in both the sham/NTHi and RV/NTHi groups 24 h post-NTHi infection, but the latter group showed comparatively less inflammation (Figure 2A and 2C). While sham/NTHi-infected animals resolved inflammation completely by 3 days post-NTHi infection, mice in the RV/NTHi group showed a persistence of neutrophilic inflammation (Figure 2B and 2D). RV/NTHi-infected mice showed a resolution of lung inflammation 7 days post-NTHi infection, correlating with clearance of bacteria from the lungs (Figure 2E).

Next we measured the levels of neutrophil-attracting chemokines in the lung homogenate supernatants. The RV/NTHi group showed a significant reduction in chemokine levels of KC/CXCL1 and MIP-2/CXCL2 compared to the sham/NTHi group (Figure 2F and 2G) at 6 and 24 h post-NTHi infection. However at 72 h post-NTHi infection, KC and MIP-2 levels were higher in the RV/NTHi group than the sham/NTHi group. Both KC and MIP-2 levels returned to normal levels by 7 days in both the sham/NTHi and RV/NTHi groups. These results imply that RV infection may suppress initial chemokine responses to subsequent bacterial challenge thereby reducing the neutrophil infiltration required for optimal bacterial clearance.

### RV infection suppresses NTHi-stimulated IL-8 in bronchial epithelial cells

**Figure 3:**
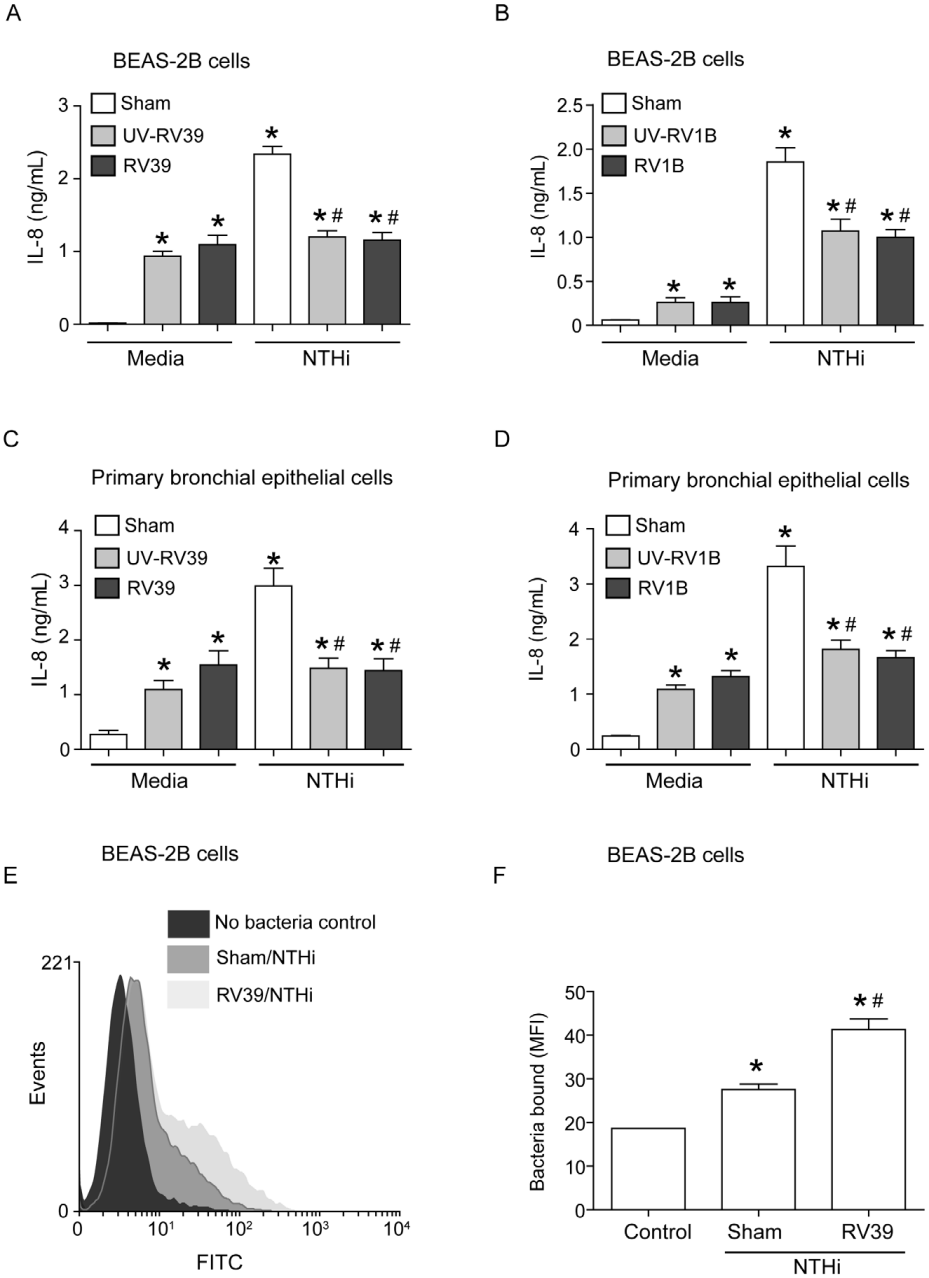
RV infection alters NTHi-stimulated IL-8 production and bacterial binding to airway epithelial cells. (A and B) BEAS-2B cells grown to 90% confluence were infected with sham, or RV39, UV-RV39, RV1B, or UV-RV1B at MOI of 1 and incubated at 33°C for 90 min. Infection media was replaced with fresh media, and incubation continued for another 22 h. Cells were then infected with NTHi (10 MOI) or treated with media, incubated for 3 h at 37°C and IL-8 in the media was assayed for IL-8 protein by ELISA. (C and D) Mucociliary-differentiated airway epithelial cells were infected apically with sham, or RV39, UV-RV39, RV1B, or UV-RV1B at MOI and incubated for 24 h. Cells were then infected with NTHi at MOI of 10 and IL-8 in the basolateral medium was determined. (E and F) BEAS-2B cells infected with RV39 or sham were infected with FITC-conjugated NTHi at MOI of 10, incubated for 1 h at 37°C, washed to remove unbound bacteria and analyzed by flow cytometry to determine binding of bacteria to cells. E and F shows representative histograms and mean fluorescence intensity respectively. Data represents mean ± SEM from 2–3 experiments performed in duplicate or triplicates (* p≤0.05, ANOVA, different from sham; #p≤0.05, ANOVA, different from sham/NTHi).

To understand the underlying mechanisms by which RV suppresses chemokine responses to subsequent NTHi infection, we performed *in vitro* studies using bronchial epithelial cells and confirmed key results in mouse alveolar macrophages. A human bronchial epithelial cell line (BEAS-2B cells) was infected with RV39 (a major group RV), RV1B or sham. Cells were then infected with NTHi and IL-8 protein in the cell culture supernatants was measured (Figure 3A and 3B). As observed previously, infection with RV or UV-RV stimulated IL-8 production in airway epithelial cells [25]. There was no difference between cells treated with RV and UV-RV. We have previously shown that RV binding/endocytosis is sufficient for stimulation of IL-8 expression in airway epithelial cells during the early phase of infection [25]. Cells infected with sham followed by NTHi showed significantly higher IL-8 levels compared to cells infected with sham or RV alone. In contrast, cells infected with RV39 or RV1B followed by NTHi showed IL-8 levels that were not significantly different from cells infected with RV alone. RV/NTHi-infected cells also showed significantly less IL-8 than sham/NTHi-infected cells, indicating that prior infection with RV suppresses NTHi-induced IL-8 production. Replication-deficient UV-irradiated RV had a similar effect. This was not a result of increased cell death, as there was no difference in LDH release or Annexin V/propidium iodide staining between sham/NTHi- and RV/NTHi-infected cells (data not shown).

To examine whether RV infection suppresses NTHi-induced IL-8 expression in well-differentiated primary airway epithelial cells, we infected these cells apically with RV39 or RV1B, incubated for 24 h and then infected with NTHi. Levels of IL-8 in the basolateral media were determined 6 h after NTHi infection. RV/NTHi-infected cells showed lower levels of IL-8 than the sham/NTHi-infected cells indicating that RV suppresses NTHi-stimulated IL-8 in primary cells (Figure 3C and 3D). Cells infected with UV-irradiated RV39 or RV1B also suppressed the IL-8 response to subsequent NTHi infection.

### Pre-infection with RV, does not affect NTHi binding to bronchial epithelial cells

To investigate whether the suppressed IL-8 response to NTHi in RV-infected bronchial epithelial cells was due to decreased NTHi binding, we determined the binding of FITC-labeled NTHi to sham- or RV39-infected cells. Compared to uninfected cells, both sham/NTHi and RV39/NTHi infected cells showed a significant shift of histogram to the right and increased mean fluorescence intensity (MFI) indicating NTHi binding (Figure 3E and Figure 3F). Compared to sham/NTHi infected cells, RV39/NTHi infected cells showed further rightward shift of histogram and increased MFI, implying that prior infection with RV39 increases binding of bacteria to the cells. These results indicate that the suppressed IL-8 response in RV/NTHi-infected cells is not due to decreased bacterial binding.

### RV infection reduces IL-8 responses to subsequent challenge with TLR agonist

**Figure 4:**
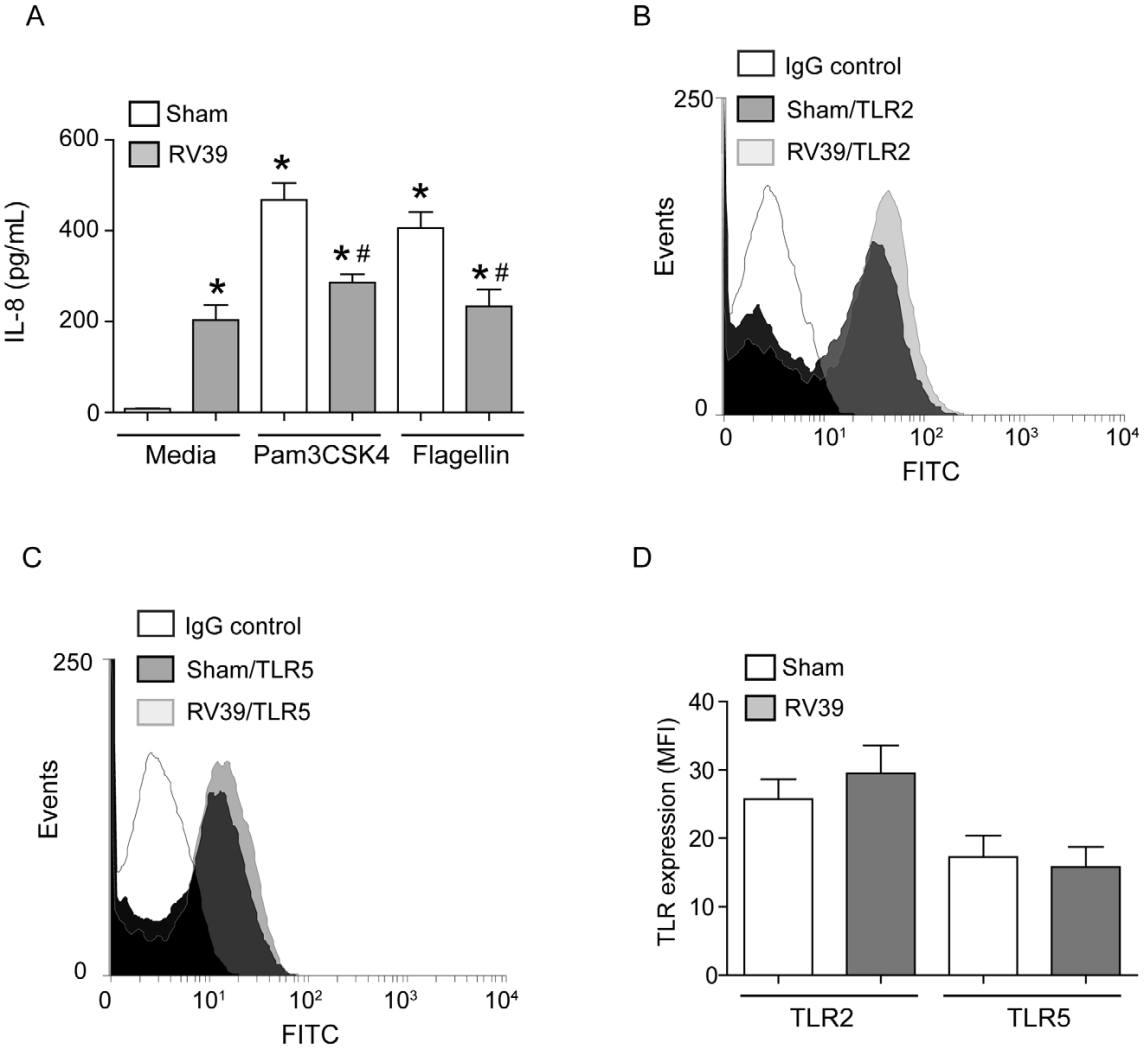
RV suppresses TLR2 or TLR5 ligands-elicited IL-8 production without affecting TLR2 or TLR5 expression. (A) BEAS-2B cells were infected with RV39 or sham as described under figure 2 and then treated with Pam3CSK4 (TLR2 agonist), or flagellin (TLR5 agonist) and incubated for 6 h. IL-8 protein levels in the cell culture supernatants were determined by ELISA. (B, C and D) BEAS-2B cells were infected with RV39 or sham, blocked and incubated with antibodies to TLR2 or TLR5 followed second antibody conjugated with Alexafluor-488 and analyzed by flow cytometry. Histograms are representative of three independent experiments. Data in A and D represents mean ± SEM calculated from 3 independent experiments performed in duplicate (* p≤0.05, ANOVA, different from sham; #, p≤0.05, ANOVA, different from sham/TLR2 or sham/TLR5 treated cells).

Bacterial recognition by TLR2 and 5 contributes to IL-8 production in bronchial epithelial cells [26]. Therefore we examined whether RV suppresses TLR2- or TLR5-induced IL-8 responses in bronchial epithelial cells. BEAS-2B cells infected with sham and then challenged with a TLR2 (Pam3CSK4) or TLR5 (flagellin) agonist showed significant increases in IL-8 levels compared to sham alone infected cells (Figure 4A). Cells infected with RV39 followed by TLR2 or TLR5 challenge showed significantly lower IL-8 responses compared to similarly-challenged sham-infected cells.

### TLR2 and TLR5 expression is not decreased by RV infection

RV-induced reductions in TLR-mediated IL-8 expression may relate to either reduced expression of TLRs or disruption of MyD88-dependent signaling, which is common for both TLR2 and 5. To assess the effect of RV infection on TLR expression, sham- or RV-infected cells were incubated with normal IgG or antibodies specific to TLR2 or TLR5 and analyzed by flow cytometry. BEAS-2B cells expressed both TLR2 and 5 on their surface, which were not significantly altered in RV39-infected cells (Figure 4B to 4D). These results suggest that suppression of TLR2 or TLR5 agonist-stimulated IL-8 in RV39-infected cells is not due to reduced TLR expression.

### IRAK-M levels are enhanced in RV-infected cells

**Figure 5:**
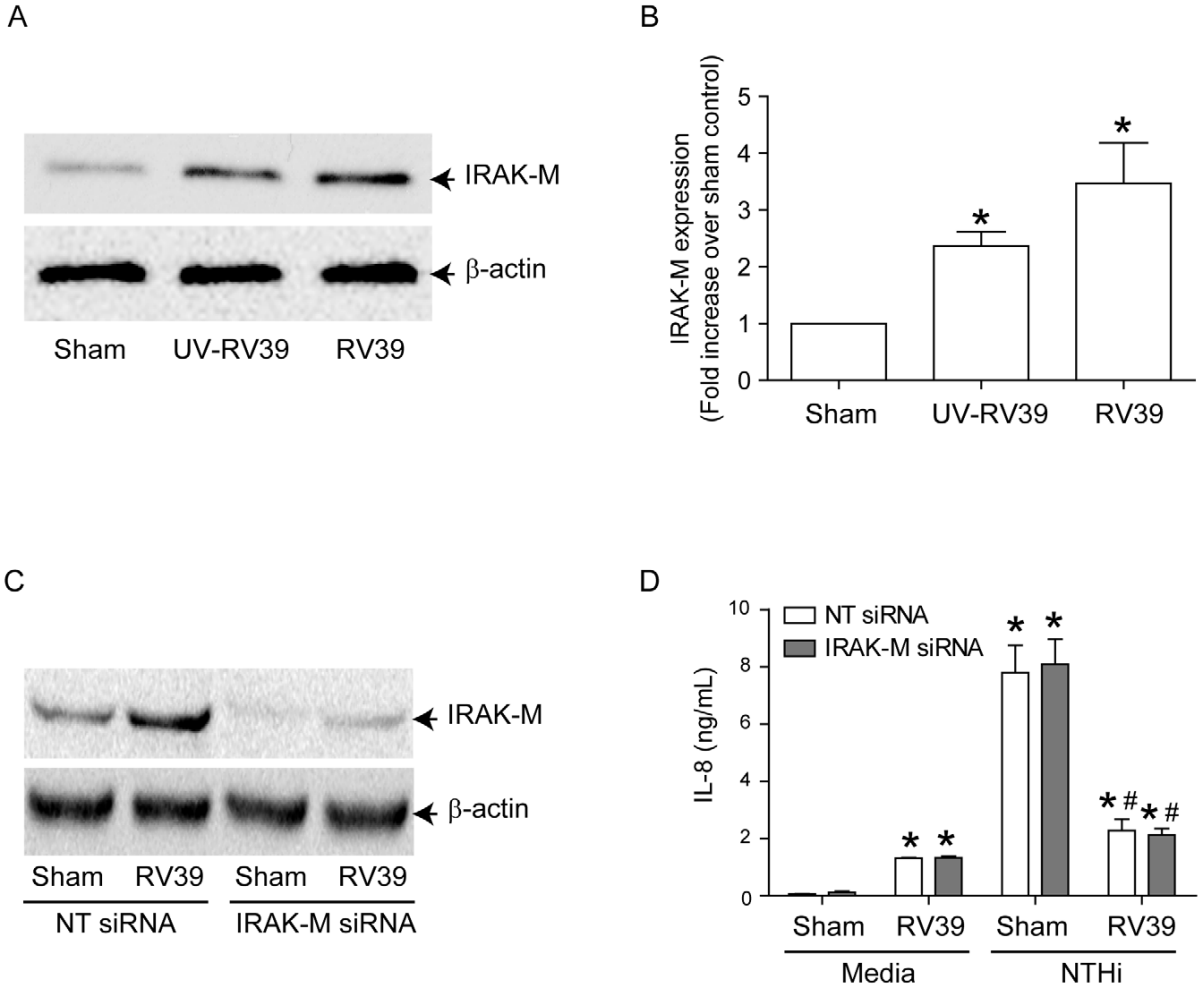
IRAK-M is not responsible for RV-induced suppression of NTHi-stimulated IL-8 in airway epithelial cells. (A and B) BEAS-2B cells were infected with sham, RV39 or UV-RV39 as described under Figure 2. Aliquots of total cell lysates corresponding to equal amount of protein from were subjected to Western blot analysis with IRAK-M antibody. (A) Representative blot showing IRAK-M expression. (B) Band intensities were quantified by NIH image and levels of IRAK-M was normalized to β-actin and expressed as fold increase over sham infected cells. (C) BEAS-2B cells were transfected with non-targeting (NT)- or IRAK-M siRNA and knockdown of IRAK-M was confirmed by Western blot analysis. (D) Similarly transfected cells were infected with sham or RV followed by NTHi as described in Figure 3 and IL-8 determined in the media. Data represents mean±SEM calculated from 3 independent experiments performed in triplicate (* p≤0.05, ANOVA, different from sham; #p≤0.05, ANOVA, different from sham/NTHi).

IRAK-M (also known as IRAK-3) is a well-described inhibitor of MyD88-dependent TLR signaling [27]. To investigate the possible role of IRAK-M in the suppression of NTHi-, or TLR2- or TLR5 agonist-induced IL-8, IRAK-M expression was assessed in RV39-infected BEAS-2B cells. RV significantly increased IRAK-M levels compared to sham controls (Figure 5A and 5B), suggesting that IRAK-M may be responsible for the observed suppression of NTHi- or TLR2 or 5 agonist-induced IL-8 expression in RV- infected cells. To examine this possibility, we transfected BEAS-2B cells with non-targeting or IRAK-M siRNA and then infected with either sham or RV. Western blot analysis showed that compared to NT–siRNA, IRAK-M siRNA transfection reduced IRAK-M expression by 83 and 77% in sham- and RV-infected cells respectively, an indication of efficient knockdown of IRAK-M in these cells (Figure 5C). However, knockdown of IRAK-M did not reverse the suppression of NTHi-stimulated IL-8 in RV-infected cells (Figure 5D). Similar results were observed when cells were challenged with TLR2 or TLR5 agonists instead of infecting with NTHi (data not shown). These data suggest that although RV increases IRAK-M expression, factors other than IRAK-M contribute to RV-induced suppression of NTHi-stimulated IL-8 response in bronchial epithelial cells.

### IRAK-1 expression is reduced in RV infection

**Figure 6:**
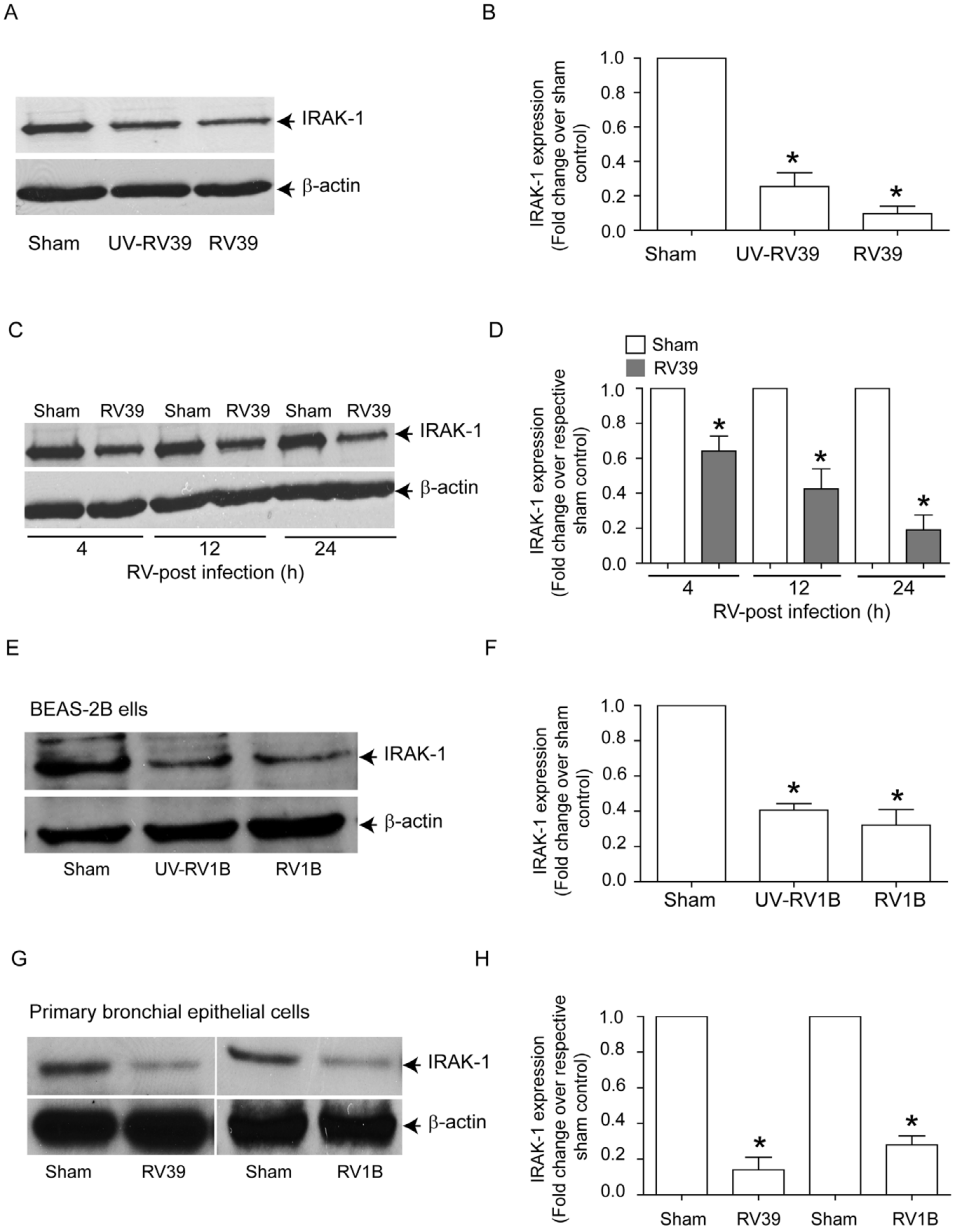
RV infection decreases IRAK-1 protein levels in both BEAS-2B and primary airway epithelial cells. (A and B) BEAS-2B cells were infected with sham, RV39, or UV-RV39, incubated for 24 h and the cells were lysed in RIPA buffer. The cell lysates containing equal amounts of protein was subjected to Western blot analysis with IRAK-1 antibody. (C and D) BEAS-2B cells were infected with sham or RV39 and cells were lysed at 4, 12 and 24 h post-infection and the lysates subjected to Western blot analysis with IRAK-1 antibody. (E and F) BEAS-2B cells infected with sham, UV-RV1B or RV1B were examined for IRAK-1 levels 24 h post- infection by Western blot analysis. (G and H) Mucociliary differentiated cells were infected apically with sham, RV39 or RV1B and IRAK-1 levels were determined 24 h later by Western blot analysis. Images are representative of 2–4 independent experiments. (B, D, F and H) Band intensities of IRAK-1 were normalized to β-actin and expressed as fold change over respective sham control. Data represents mean ± SEM calculated from 3 to 4 independent experiments (* p≤0.05, ANOVA, different from sham).

It was recently shown that IL-1β and MyD88 are partially required for RV-induced IL-8 expression in BEAS-2B cells [28]. IRAK-1 undergoes proteosomal degradation following activation of the IL-1/TLR signaling pathway, thereby inducing a state of tolerance to subsequent TLR stimulation [29]. It is therefore conceivable that RV infection induces degradation of IRAK-1, leading to the attenuation of TLR-mediated IL-8 production, irrespective of IRAK-M status. To test this hypothesis, we determined the levels of IRAK-1 in BEAS-2B cells infected with either sham, RV39 or UV-irradiated RV39. Infection with RV39 and UV-RV39 each decreased IRAK-1 protein levels significantly 24 h after incubation (Figure 6A and 6B). RV39 infection decreased IRAK-1 levels as early as 4 h post-infection (Figure 6C and 6D). UV-RV39 also reduced IRAK-1 protein levels, indicating that the changes in IRAK-1 protein abundance do not require viral replication. Similar decreases in IRAK-1 were observed in BEAS-2B cells infected with RV1B (Figure 6E and 6F) and primary airway epithelial cells infected with RV39 or RV1B (Figure 6G and 6H).

**Figure 7:**
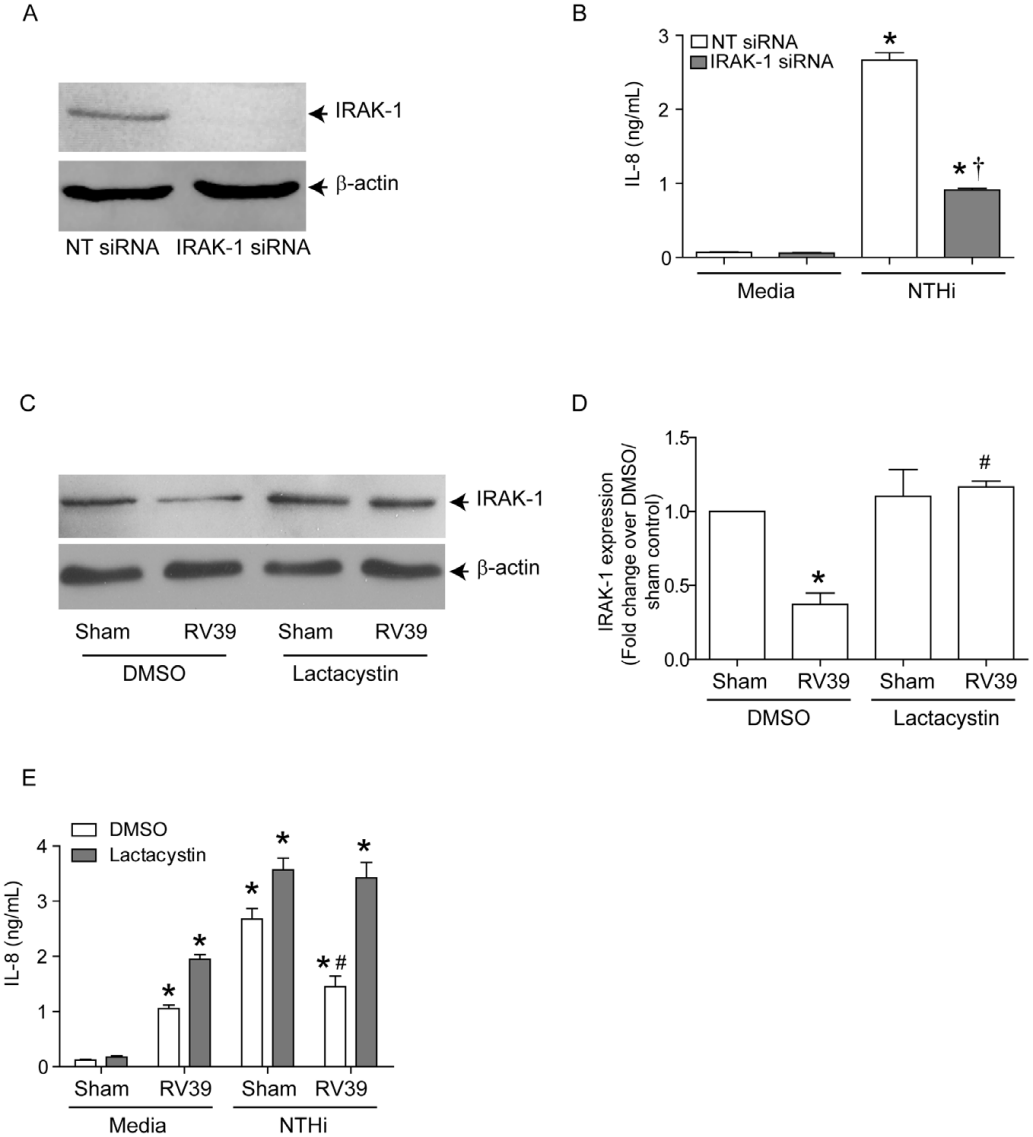
IRAK-1 is required for NTHi-stimulated IL-8 production. BEAS-2B cells were transfected with non-targeting (NT)- or IRAK-1 siRNA and grown for 3 days. (A) Cell lysates were prepared from transfected cells and subjected to Western blot analysis to confirm knockdown of IRAK-1. (B) Transfected cells were infected with NTHi, incubated for 3 h and IL-8 in the media determined. BEAS-2B cells were infected with sham or RV39 in the presence or absence of 5 µM lactacystin and incubated for 24 h. (C and D) Cells were either harvested for determination of IRAK-1 expression by Western blot analysis and band intensities of IRAK-1 were normalized to β-actin. (E) Cells were then infected with NTHi and IL-8 in the media was examined. Western blot images are representative of 3 experiments. Data in B, D and E represents mean±SEM calculated from 3 independent experiments performed in triplicate (* p≤0.05, ANOVA, different from sham; †, p≤0.05, ANOVA different from NT-siRNA transfected cells; # p≤0.05, ANOVA, different from DMSO treated RV/NTHi-infected cells).

To determine whether IRAK-1 is required for NTHi-induced IL-8 expression, BEAS-2B cells transfected with non-targeting or IRAK-1 siRNA were infected with NTHi and the IL-8 response was determined 3 h later. Immunoblotting showed complete knockdown of IRAK-1 in cells transfected with IRAK-1 but not in non-targeting siRNA (Figure 7A). NTHi-induced IL-8 was significantly reduced in cells transfected with IRAK-1 siRNA compared to NT-siRNA-transfected cells (Figure 7B). These results imply that IRAK-1 is required for NTHi-induced IL-8 expression, consistent with the notion that reduced IRAK-1 protein levels contribute to RV-induced suppression of NTHi-induced IL-8 expression.

To determine whether inhibition of IRAK-1 degradation in RV-infected cells restores NTHi-stimulated IL-8 expression, we infected cells with RV39 in the presence of proteosomal inhibitor lactacystin and examined IRAK-1 and IL-8 levels. In DMSO-treated cells (vehicle control), RV39 infection decreased IRAK-1 protein levels significantly (Figure 7C and 7D) and also suppressed NTHi-stimulated IL-8 (Figure 7E). By contrast, pre-treatment with lactacystin (5 µM) prevented RV-mediated reductions in IRAK-1 protein abundance and NTHi-induced IL-8 expression. These results show that IRAK-1 degradation caused by RV contributes to the reduced IL-8 response to subsequent NTHi infection.

### IL-1 receptor antagonist (IL-1ra) does not reverse suppression of NTHi-stimulated IL-8 in RV infected cells

Since RV has been shown to activate MyD88-dependent signaling via IL-1β [28], we hypothesized that IL-1β is required for the suppression of NTHi-stimulated IL-8 in RV-infected cells. To test this hypothesis, BEAS-2B cells were infected with sham or RV in the presence or absence of IL-1ra. Cells were then infected with NTHi and IL-8 measured in the cell culture supernatant. IL-1ra did not reverse the suppression of NTHi-stimulated IL-8 caused by RV, even at high concentration (Supplemental Figure S2A). In addition, IL-1ra failed to restore the levels of IRAK-1 in RV-alone infected cells (Supplemental Figure S2B). Together, these results suggest that RV-induced IRAK-1 degradation is triggered independently of IL-1β.

### RV-induced TLR2 activation is sufficient for reduction in IRAK-1 protein levels

**Figure 8:**
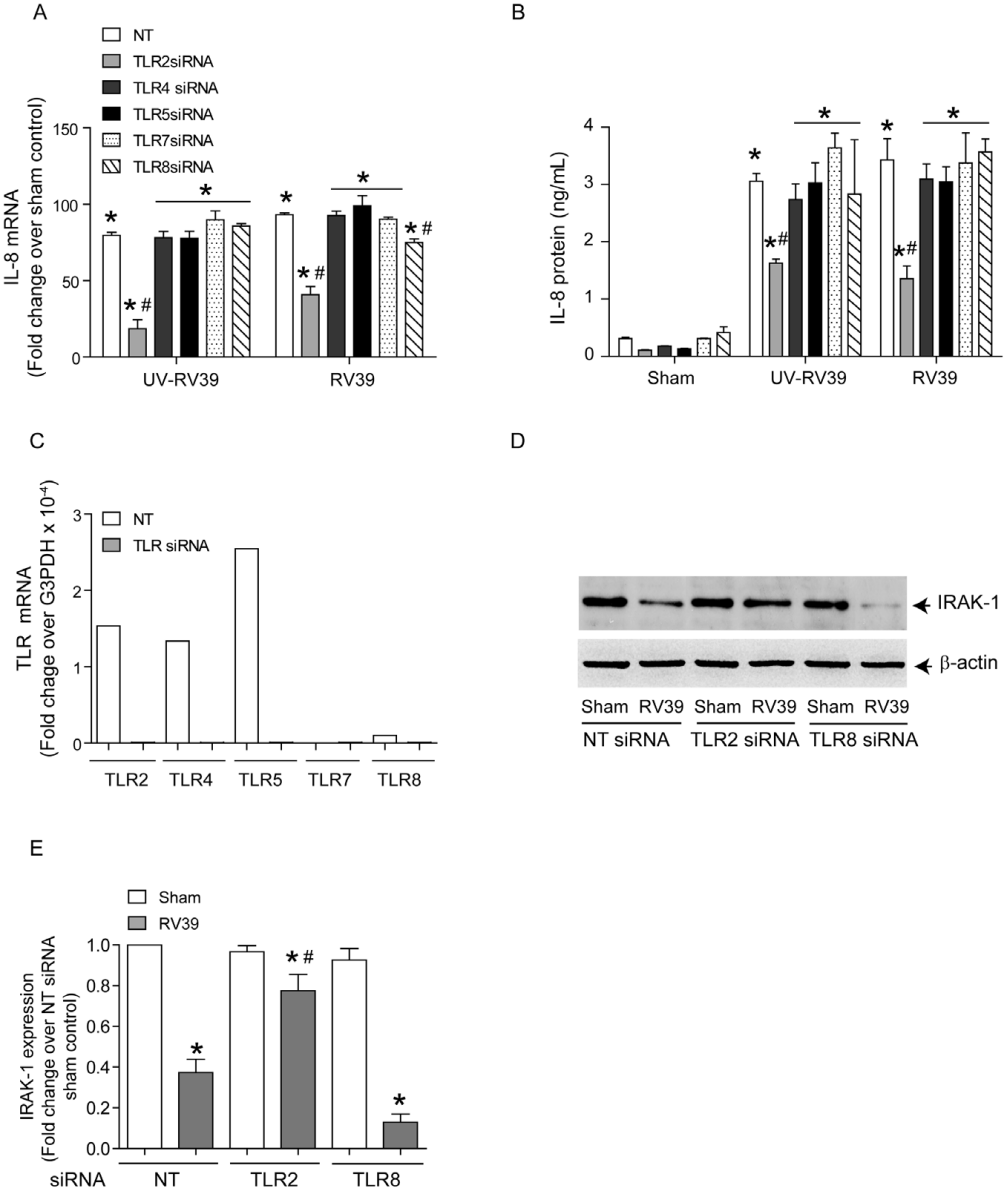
RV-decreases IRAK-1 protein via TLR2 activation. BEAS-2B cells were transfected with NT, TLR2, TLR4, TLR5, TLR7 or TLR8 siRNA and grown for 2 days. (A and B) Cells were infected with sham, UV-RV39 or RV39 and incubated for 24 h. mRNA and protein levels of IL-8 was determined by qPCR and ELISA respectively. (C) Knockdown of TLRs was confirmed by qPCR. Data is normalized to GAPDH and is representative of three independent experiments. (D) Cells transfected with NT-, TLR2 or TLR8 siRNA were infected with sham or RV39 as above and cell lysates subjected to Western blot analysis with IRAK-1 antibody. (E) Band intensities of IRAK-1 were normalized to β-actin and expressed as fold increase over respective NT/sham control. Image in D is representative of 3 independent experiments. Data in A, B and E represents mean±SEM calculated from 3 independent experiments performed in triplicate (* p≤0.05, ANOVA, different from sham; # p≤0.05, ANOVA, different from NT-siRNA transfected cells).

We examined the contribution of the MyD88-dependent TLRs TLR2, −4, −5, −7 and −8 to RV-induced IL-8 expression. BEAS-2B cells were transfected with non-targeting siRNA or gene specific siRNA to TLR2, TLR4, TLR5, TLR7 or TLR8 and infected with RV39. IL-8 mRNA and protein levels were determined 24 h post-infection. TLR2 siRNA-transfected cells infected with UV-RV or RV showed significantly lower IL-8 mRNA and protein than similarly-infected non-targeting siRNA-transfected cells (Figures 8A and 8B). TLR8 siRNA-transfected cells showed a small (20%) but significant reduction in RV-induced IL-8 mRNA, but not protein levels. Knockdown of TLR4 TLR5, or TLR7 had no effect on RV or UV-RV-induced IL-8 responses either at the mRNA or at protein levels. Next we assessed the knockdown of each TLR by quantifying mRNA by qPCR (Figure 8C) and protein by flow cytometry (Supplemental Figure S3). BEAS-2B cells showed expression of TLR 2, −4, and −5 at both the mRNA and protein level, and transfection of gene-specific siRNA knocked down the expression. BEAS-2B cells did not express TLR7. Cells expressed TLR8 at very low levels and expression was completely inhibited in cells transfected with TLR8 siRNA.

Since only knockdown of TLR2 and TLR8 reduced RV-induced IL-8 responses, we examined the levels of IRAK-1 protein in TLR2 or TLR8 siRNA-transfected cells after RV infection. Compared to non-targeting siRNA-transfected cells infected with RV39, similarly-infected TLR2 siRNA-transfected cells showed IRAK-1 levels similar to sham-infected cells (Figure 8D and 8E). In contrast TLR8 siRNA transfected cells infected with RV showed IRAK-1 degradation similar to cells transfected with NT siRNA. These results imply that TLR2, but not TLR8 is required for the RV-induced reduction in IRAK-1 protein levels.

To assess whether TLR2 or TLR8 knockdown restores NTHi-stimulated IL-8 expression in RV-infected cells, cells transfected with non-targeting, TLR2 or TLR8 siRNA were infected with sham or RV39 and then infected with NTHi. Not surprisingly, TLR2 knockdown reduced NTHi-stimulated IL-8 (Supplemental Figure S4), as NTHi-induced IL-8 expression is TLR2-dependent [30]. On the other hand, TLR8 knockdown had no effect on NTHi-stimulated IL-8 and did not reverse the suppressive effect of RV on NTHi-stimulated IL-8.

### Alveolar macrophages infected with RV1B show decreased IRAK-1 expression

**Figure 9:**
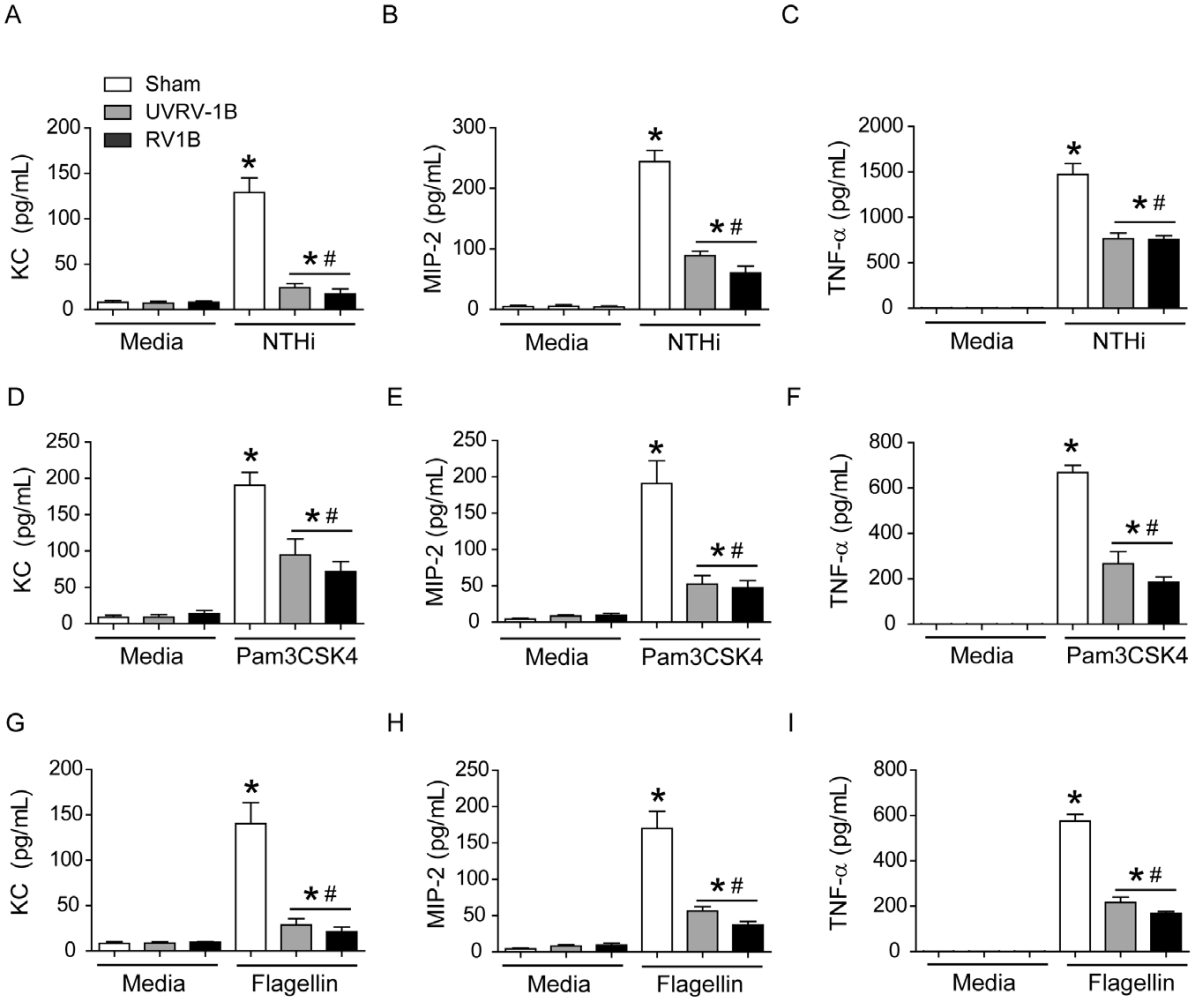
RV infection suppresses NTHi- and TLR-stimulated chemokine and cytokine responses in alveolar macrophage. MH-S cells were infected with sham, RV1B or UV-RV1B. Cells were then challenged with (A to C) NTHi, (D to F) Pam3CSK4 or (G to I) flagellin and protein levels of KC, MIP-2 and TNF-α in the medium was measured after 3 (for TNF-α) or 6 h (for KC and MIP-2) post challenge by ELISA. Data represent mean±SEM calculated from 3 independent experiments performed in duplicates (* p≤0.05, ANOVA, different from sham; # p≤0.05, ANOVA, different from sham/NTHi infected cells).

**Figure 10:**
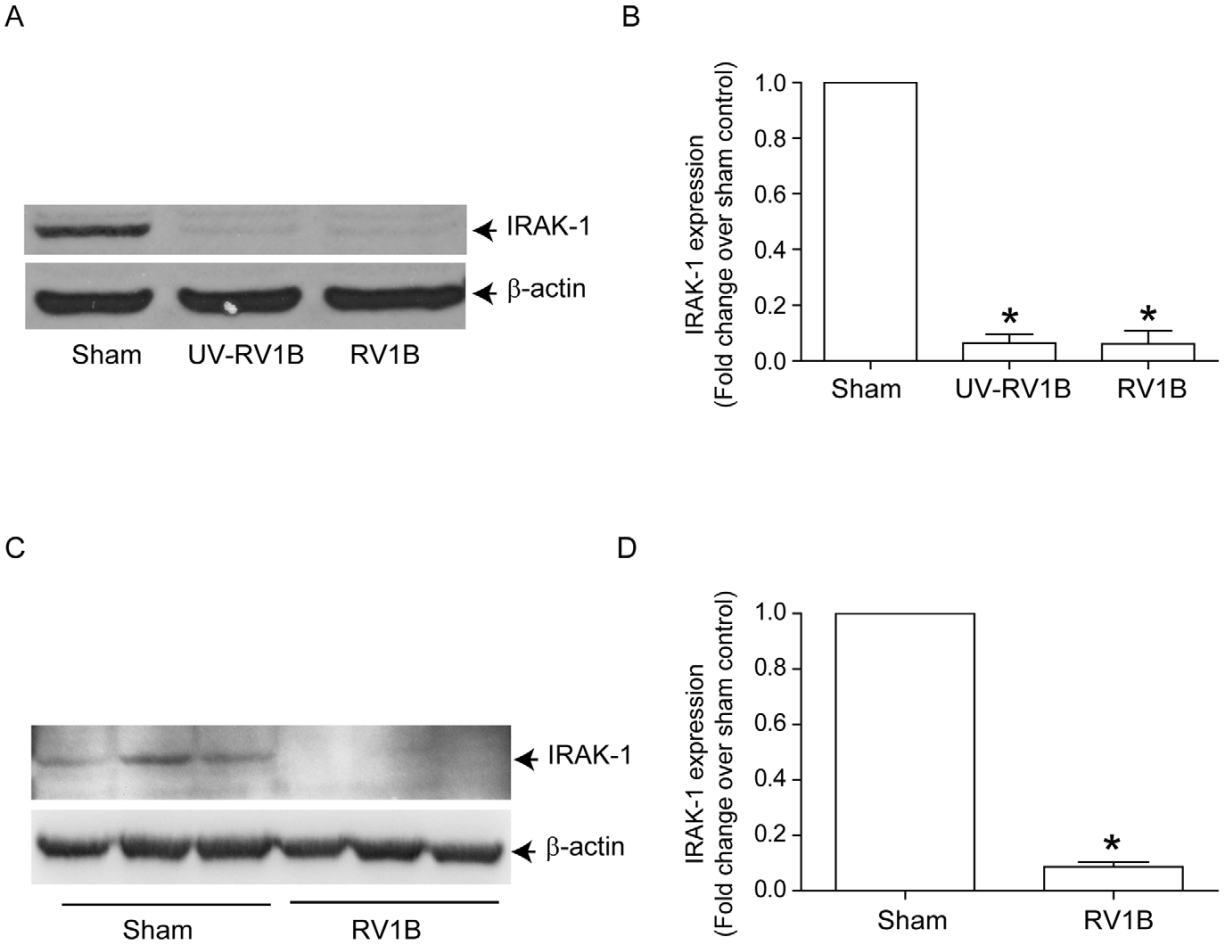
RV infection decreases IRAK-1 expression in alveolar macrophages *in vitro* and in mice lungs *in vivo*. (A and B) MH-S cells were infected with sham, RV1B or UV-RV1B as described in figure 2 and incubated for 24 h. Cells were lysed in RIPA buffer and aliquots of cell lysates containing equal amounts of protein was subjected to Western blot analysis with IRAK-1 antibody. (C and D) Mice were infected with sham or RV1B, sacrificed two days later and lung lysates subjected to Western blot analysis to determine IRAK-1 expression. (A and C) Image presented is a representative of three independent experiments and 3 different animals respectively. (B and D) Band intensities of IRAK-1 were normalized to β-actin and expressed as fold increase over sham control. Data represents mean and SEM calculated from 3 independent experiments in B and 4 animals in D (* p≤0.05, ANOVA, different from sham).

RV infection has been shown to desensitize TLR4-dependent signaling in alveolar macrophages [22]. We therefore assessed the effects of RV1B infection on NTHi-stimulated cytokine expression and IRAK-1 protein levels in MH-S mouse alveolar macrophages. MH-S cells were infected with sham, UV-RV1B or RV1B and incubated for 24 h. Cells were then infected with NTHi and levels of KC, MIP-2 and TNF-α in the medium were determined 6 h later. Both UV-RV1B/NTHi and RV1B/NTHi-infected cells showed significantly less KC, MIP-2 and TNF-α than the sham/NTHi-infected cells (Figure 9A–9C). On a similar note, RV1B-infected cells also showed decreased KC and TNF-α production in response to secondary challenge with TLR2 (Figure 9D–9F) or TLR5 agonists (Figure 9G–9I). Compared to sham-, UV-RV1B and RV1B-infected cells also showed reduced IRAK-1 protein levels (Figure 10A and 10B), indicating that RV-induced IRAK-1 degradation may be responsible for reduced cytokine production in alveolar macrophages stimulated with bacteria or TLR agonists.

#### RV1B infection reduces IRAK-1 protein levels *in vivo*

Next, we assessed whether RV infection affects IRAK-1 protein levels *in vivo*. Mice were infected with sham or RV1B and 48 h later, IRAK-1 protein was determined in the whole lung homogenates by Western blot analysis. Compared to sham infected mice, RV1B infected mice showed a significant reduction in IRAK-1 levels (Figure 10C and 10D). Together, these results indicate that RV-induced reductions in IRAK-1 may attenuate chemokine production in response to secondary bacterial infection via desensitization of MyD88-dependent TLR signaling.

## Discussion

In this study, we demonstrate for the first time that RV infection delays bacterial clearance *in vivo*. Delayed bacterial clearance was accompanied by attenuated expression of the neutrophil-attracting chemokines KC/CXCL1 and MIP-2/CXCL2. We also show that, despite increasing bacterial binding, RV suppresses chemokine responses to subsequent NTHi infection in cultured bronchial epithelial cells. RV infection also attenuated TLR2- and TLR5-stimulated chemokine responses in cultured bronchial epithelial cells and alveolar macrophages, suggesting that RV infection interferes with TLR-dependent innate immune defenses. Most importantly, we define a mechanism by which RV infection desensitizes TLR signaling in both bronchial epithelial cells and alveolar macrophages. We demonstrate that RV infection causes degradation of IRAK-1, a key adaptor protein in MyD88-dependent TLR signaling, thereby suppressing chemokine responses to subsequent NTHi challenge. Further, we show that IRAK-1 degradation was dependent on TLR2, but not on other TLRs which activate MyD88-dependent signaling. Based on these observations, we speculate that suppression of TLR-dependent innate immune responses increases the risk of acquiring secondary bacterial infection and promotes invasiveness of bacterial flora that is present in the nasopharynx/lower airways of CF and COPD patients.

Although there is clinical evidence indicating that RV infection precedes bacterial infection-associated disease exacerbations [3], [4], [5], until now there were no experimental data demonstrating an increased risk of bacterial infection following RV infection *in vivo*. Using a novel mouse model of RV infection that we developed [24], [31], we found that RV infection promotes NTHi persistence in the lungs, likely by attenuating KC and MIP-2 expression and impairing recruitment of neutrophils to the airways. Appropriate neutrophil recruitment is required for bacterial clearance, particularly under circumstances that exceed the antimicrobial capacity of airway epithelial cells and alveolar macrophages. Mice with a delayed neutrophil response fail to clear *P. aeruginosa* efficiently from their lungs [32]. Recently, influenza virus was also shown to suppress KC and MIP-2 responses to subsequent bacterial infection; suppression was dependent on virus-stimulated type I interferons [15]. Since RV infection also stimulates type I interferon responses both *in vivo* and in airway epithelial cells *in vitro* [24], [33], [34], [35], [36], it is plausible that RV suppresses chemokine responses via this mechanism. However, this is unlikely, because replication-deficient UV-irradiated RV, which does not stimulate expression of type I interferons, also attenuated chemokine response to NTHi infection *in vitro*.

Both bronchial epithelial cells and alveolar macrophages express TLRs. TLRs play a crucial role in the establishment of appropriate innate immune responses, including expression of chemokines, upon recognition of pathogen associated molecular patterns. Previously, RV infection was shown to suppress subsequent cytokine responses to LPS, a TLR4 ligand in human alveolar macrophages [22]. Suppression was dependent on viral replication. In contrast, we found that attenuation of TLR-dependent signaling in both bronchial epithelial cells and alveolar macrophages was independent of viral replication. These data suggest that suppression is initiated by an early event in the viral life cycle, for example, binding or endocytosis. This is not surprising, because RV binding itself has been shown to activate signaling mechanisms leading to phosphatidylinositol 3-kinase activation, IL-8 expression and generation of reactive oxygen species [25], [37].

In the present study, we found that RV infection not only attenuated chemokine expression due to subsequent challenge with NTHi infection, but also to TLR2 and TLR5 ligands. NTHi stimulates TLR2 signaling leading to pro-inflammatory cytokine expression [30]. In addition, both TLR2 and TLR5 initiate a MyD88-dependent signaling pathway leading to chemokine and pro-inflammatory cytokine expression. We therefore hypothesized that RV attenuates MyD88-dependent TLR signaling. Accordingly, we found that RV infection significantly increased IRAK-M while dramatically reducing the levels of IRAK-1 protein in both airway epithelial cells and murine alveolar macrophages. IRAK-M is thought to be a negative regulator of MyD88-dependent TLR signaling [38]. However, genetic silencing of IRAK-M did not restore NTHi-induced chemokine expression in RV-infected airway epithelial cells, suggesting that IRAK-M does not contribute to RV-induced attenuation of IL-8 expression. This is not entirely unexpected, as IRAK-M has been demonstrated to play a vital role in the suppression of a non-canonical TLR signaling pathway that relies on activation of NF-κB-inducing kinase rather than IκB kinase [39].

In contrast, blocking of RV-induced reduction in IRAK-1 by using a proteasomal inhibitor restored NTHi-induced IL-8 responses in RV-infected bronchial epithelial cells, indicating a role for IRAK-1. Previously, it has been shown that post-translational modification of IRAK-1, such as hyperphosphorylation and ubiquitination that occurs following MyD88-dependent TLR or IL-1 signaling, leads to IRAK-1 degradation [29]. IRAK-1 degradation constitutes a mechanism for limiting exaggerated innate immune responses. In the present study, we found that, *in vitro*, IRAK-1 is degraded as early as 4 h post-RV infection and the IRAK-1 protein levels remain low for at least 24 h. Pre-treatment with lactacystin prevented reductions in IRAK-1 protein abundance, suggesting that RV promotes proteasomal degradation of IRAK-1. Reduced IRAK-1 was also observed in mice infected with RV 48 h post-infection. These observations suggest that RV induces IRAK-1 degradation via the activation of MyD88-dependent IL-1/TLR signaling cascades. However, since lactacystin can also inhibit proteasomal degradation of other proteins of TLR pathway, further studies using IRAK-1 mutants which are not amenable to ubiquitation and proteasomal degradation are necessary to further confirm the specific role of IRAK-1 in RV-induced suppression of NTHi-stimulated chemokine responses.

Recently, RV infection of BEAS-2B cells was shown to elicit low levels of IL-1β release, which in turn induced MyD88-dependent potentiation of IL-8 production [28]. However, in the present study, inhibition of IL-1 receptor by IL-1ra neither blocked RV-induced degradation of IRAK-1 nor reversed suppression of NTHi-stimulated IL-8, suggesting that the IRAK-1 degradation that we observed is not due to RV-induced IL-1β signaling.

RV single-stranded RNA may activate MyD88-dependent TLR signaling directly via TLR7 and TLR8. In our studies we found that airway epithelial cells do not express TLR7 and express TLR8 at very low levels. Further, knockdown of TLR8 neither prevented RV-induced degradation of IRAK-1 nor reversed suppressive effects of RV on NTHi-stimulated IL-8. Similar to our results, recently it TLR7/8 agonist, imiquimod was found to be inefficient in activating airway epithelial cells suggesting that these cells may not express TLR7 or 8 [40]. On the other hand, it has been demonstrated that both intact and UV-irradiated RV-6 stimulate NF-κB transactivation in TLR2-transfected HEK-293 cells [41]. Since we observed that both replication-competent and replication-deficient RV were equally capable of causing IRAK-1 degradation, we posit that TLR2 plays a role in RV-induced IRAK-1 degradation. Consistent with this notion, we found that knockdown of TLR2 significantly reduced RV-induced IRAK-1. However, we could not determine the effect of TLR2 knockdown on suppression of NTHi-stimulated IL-8 in RV-infected cells, because NTHi also requires TLR2 to stimulate IL-8 [30]. Also, the precise mechanism by which RV activates TLR2 remains to be elucidated.

In summary, we have shown that RV suppresses NTHi-induced IL-8 expression in airway epithelial cells and alveolar macrophages by inducing TLR2-dependent degradation of IRAK-1. Our results suggest that RV may increase the risk of acquiring secondary bacterial infection by attenuating TLR-dependent innate immune responses.

## Materials and Methods

### Ethics statement

This study was carried out in strict accordance with the recommendations in the Guide for the Care and Use of Laboratory Animals of the National Institutes of Health, United States. The protocol was approved by the Institutional Animal Care and Use Committee of the University of Michigan Medical School. All surgery was performed under sodium pentobarbital anesthesia, and all efforts were made to minimize suffering. Tracheal and bronchial trimmings from donor lungs obtained at the time of double lung transplantation (which are otherwise discarded) were used for isolation of airway epithelial cells and the protocol was approved by the University of Michigan Investigational Review Board. Since the tissues were not collected for the purpose of isolation of cells and there was no documentation linking the tissue to the donor, the University of Michigan Investigational Review Board provided waiver for obtaining consent.

### Rhinovirus

RV39 and RV1B was obtained from ATCC and used to generate viral stocks via infection of HeLa cells as previously described [25]. Virus titer was determined by assessing 50% tissue culture infectivity dose (TCID_50_)/ml. For production of UV-irradiated (UV-RV) RV, samples were irradiated using a CL-1000 crosslinker (UVP, Upland, CA) at 10 mJ/cm^2^ for 10 min on ice as described previously [33]. RV inactivated by this method is fully capable of binding to epithelial cells and undergoing endocytosis and stimulates IL-8 protein levels similar to intact RV. At the same time it does not stimulate replication-dependent interferons and interferon-dependent genes.

### Bacterial culture and conditions

Nontypeable *Haemophilus influenzae* (6P5H) was isolated from a COPD patient during exacerbation (Kindly provided by T. Murphy, University of Buffalo, Buffalo, NY). Bacteria was maintained as a glycerol stock at −80°C and cultured as described previously [42].

### Animals and infection

Normal 8 to 10 week-old BALB/C mice were infected with 50 µl of RV1B (1×10^8^ TCID_50_T/ml) by intranasal route as described previously [24], [31]. Mice were then infected with 40 µl NTHi (1×10^9^ colony forming units/ml) by intratracheal route [43] and sacrificed 6 h, 1 day or 3 days or 7 days post-NTHi infection. Lungs were collected under aseptic conditions, then homogenized in 2 ml sterile PBS. An aliquot of lung homogenate was 10 fold serially diluted and plated on chocolate agar plates to determine bacterial density. Lung homogenates were centrifuged and supernatants used for ELISA. Number of airway and tissue inflammatory cells was determined by counting cells in bronchoalveolar (BAL) fluid and lung digests respectively as described previously [34]. Briefly, after appropriate treatment, BAL was performed by instilling 1 ml PBS containing 5 mM EDTA 10 times. Cytospins prepared from BAL cells were stained with Diff-Quick and differential counts were determined by counting a minimum of 200 cells. To quantify the number of inflammatory cells in the tissues, lungs were digested with type IV collagenase for 1 h, lung digests were strained through 70-µm nylon mesh (BD Biosciences, San Jose, CA) and centrifuged. The cell pellet was treated with RBC lysis buffer (BD Biosciences) and subjected to density gradient centrifugation on 40% Percoll (Sigma-Aldrich, St. Louis, MO) to enrich for leukocytes. The total cell count was determined on a hemocytometer. Cytospins of these tissue leukocytes were stained with Diff-Quick and examined for differential cell counts. In some experiments, after appropriate treatment, lungs were inflation fixed and embedded in paraffin. Five micron thick sections were deparaffinized and stained with hematoxylin and eosin to evaluate lung inflammation.

### Cell culture and infection

Human bronchial epithelial cell line (BEAS-2B; American Type Culture Collection, Manassas, VA) were grown in collagen coated plates using bronchial epithelial cell growth medium (BEGM) (Lonza, Walkersville, MD)) as described previously [33]. Primary airway epithelial cells from normal subjects were cultured at air/liquid interface to promote differentiation into mucociliary phenotype [33], [42], [44]. Mouse alveolar macrophages (MH-S) (ATCC) cells were grown in RPMI media amended with L-glutamine and 10% serum.

BEAS-2B or MH-S cells grown to 90% confluence were infected with RV or UV-RV at multiplicity of infection (MOI) of 1 or equal volume of sham (media from uninfected HeLa cells) and incubated for 90 min at 33°C. Infection media was replaced with fresh media and the incubation continued for another 22 h. Cells were then infected with NTHi at MOI of 10 or treated with media alone, centrifuged at 500*×*g for 5 min and incubated at 37°C for 3 h. Media was collected for determination of IL-8 (for BEAS-2B cells) or KC, MIP-2 and TNF-α (for MH-S cells). In some experiments, cells were infected with RV in the presence of 5 µM lactacystin (Cayman chemicals, Ann Arbor, MI) or 10 to 100 ng/ml IL-1 receptor antagonist (PeproTech, Rocky Hills, NJ). Primary cells were infected apically with RV, UV-RV or sham at 1 MOI [45], incubated for 48 h and then cells were superinfected apically with NTHi at 10 MOI and basolateral media collected for IL-8 and cells for Western blot analysis.

### TLR agonists

BEAS-2B cells were infected with RV, UV-RV or sham as described above. Cells were then challenged with TLR2 agonist, Pam3CSK4 (InvivoGen, San Diego, CA), or TLR5 agonist flagellin (ENZO Life Sciences, Inc., Farmingdale, NY), incubated for 6 h at 37°C and IL-8 determined in the cell culture supernatant.

### SDS-PAGE and Western blotting

After relevant treatment, cells were washed with ice cold PBS and total cell lysates were prepared as described previously [46]. Briefly, cells were lysed in cold RIPA buffer containing complete protease inhibitors (Roche Diagnostics, Indianapolis, IN), lysates centrifuged and total protein determined in supernatants. Lungs harvested from mice were homogenized in 1 ml PBS conataining complete protease inhibitors and mixed with 2× RIPA buffer, sonicated for 3×10 seconds each, centrifuged and total protein determined in supernatants. Aliquots equivalent to equal amounts of protein were subjected to SDS-PAGE and protein transferred to nitrocellulose membrane. Membranes were blocked and probed with antibodies to IRAK-M, IRAK-1 (both from Santa Cruz Biotechnology, Santa Cruz, CA), or β-actin (Sigma-Aldrich, St. Louis, MO). Bound antibody was detected by using appropriate second antibody conjugated with horseradish peroxidase and chemiluminescent substrate. Band densities were normalized to β-actin or relevant total protein using NIH Image-J (NIH, Bethesda, MD).

### Bacterial binding assay

Binding of bacteria to cells was determined using FITC-labeled NTHi as described previously [47]. Briefly, FITC-labeled NTHi was incubated with BEAS-2B cells infected with Sham, UV-RV or RV and incubated for 60 min. Cells were then washed and analyzed by flow cytometry (Becton-Dickinson, Franklin Lakes, NJ).

### Detection of TLR expression by flow cytometry

After relevant treatment BEAS-2B cells were incubated with blocking buffer followed by monoclonal antibodies to either TLR2, TLR4, TLR5, TLR7 or TLR8 (all from eBioscience, Inc., San Diego, CA) or normal mouse IgG (matched isotype control) conjugated with fluorescein isothiocyanate (FITC). For detection of TLR7 and TLR8 cells were permeabilized with 1% saponin for 30 min on ice prior to incubating with antibodies to TLR7 or TLR8. Cells were then washed and analyzed by flow cytometry [42].

### ELISA

Conditioned basolateral medium from cell cultures or lung homogenate supernatant was used to determine the protein levels of chemokines by ELISA (R&D systems, Minneapolis, MN) as described previously [45], [48].

### Transfection of BEAS-2B cells

BEAS-2B cells were reverse transfected with 10 picomoles of non-targeting (NT), or ON-TARGETplus SMART pool siRNA specific to TLR2, TLR4, TLR5, TLR7, TLR8, IRAK-1 or IRAK-M (Dharmacon, Inc., Chicago, IL) and incubated for 48 h. Cells were then infected with sham, RV or NTHi as appropriate (described in results section). Knockdown of gene expression was confirmed by qPCR or Western blot analysis.

### Statistics

Results are expressed as means ± SEM. Data were analyzed by SigmaStat statistical software (Systat Software, Inc., San Jose, CA). One-way analysis of variance (ANOVA) with Tukey-Kramer post-hoc analysis or two-way ANOVA was performed to compare more than two groups. To compare two groups, an unpaired t test with Welch's correction was used. If the data were not distributed normally data were expressed as range with median and analyzed by Mann-Whitney test. A value of p≥0.05 was considered significant.

## Supporting Information

Figure S1 RV/NTHi infection does not alter macrophage/monocyte and lymphocytes in mice. BALB/C mice were infected with RV1B or sham by intranasal route. Two days later, mice were infected with NTHi or treated with PBS via intratracheal route and sacrificed at 6 h, 1 day, 3 days or 7 days post-NTHi infection. Cytospins of BAL cells and leukocyte enriched fraction from lung homogenates were prepared, stained with Diffquick and number of (A and B) lymphocytes and (C and D) macrophage/monocytes were counted. Data represent mean±SD (n = 5, *p≤0.05, two-way ANOVA, different from sham-infected animals).
(TIF)

Figure S2 RV induced reduction in IRAK-1 protein is not IL-1β-dependent. BEAS-2B cells were infected with sham or RV39 and incubated at 33°C for 90 min. Infection media was replaced with media or media containing IL-1ra and incubation continued for another 22 h. (A) Cells were washed once with fresh media and then infected with NTHi and IL-8 in the media was assessed after 3 h. Data represents mean±SEM calculated from 3 independent experiments performed in triplicates (* p≤0.05, ANOVA, different from sham; #p≤0.05, ANOVA, different from sham/NTHi). (B) Cells were lysed following RV infection and the lysates corresponding to equal amounts of protein was subjected to Western blot analysis with IRAK-1 antibody. Image is a representative example of 2 independent experiments.
(TIF)

Figure S3 Gene specific siRNA to TLRs inhibits protein expression of respective TLRs. BEAS-2B cells were reverse transfected with NT-, TLR2, TLR4, TLR5, TLR7 or TLR8. After 48 h incubation, cells were either permeabilized (for TLR7 and TLR8) or not (TLR2, TLR4 and TLR5), incubated with antibodies to (A) TLR2, (B) TLR4, (C) TLR5, (D) TLR7 or (E) TLR8 or normal IgG conjugated with FITC and analyzed by flow cytometry. Image is a representative example of 2 independent experiments.
(TIF)

Figure S4 Knockdown of TLR2 decreases NTHi-stimulated IL-8. BEAS-2B cells reverse transfected with NT- or TLR2 siRNA were grown for 2 days. Cells were infected with NTHi or sham infected with media, incubated for 3 h at 37°C and IL-8 the medium was determined. Data represents mean±SEM calculated from 3 independent experiments performed in triplicates (* p≤0.05, ANOVA, different from media control; #p≤0.05, ANOVA, different from cells transfected with NT siRNA and then infected with NTHi).
(TIF)
