# Supplementary figures and images for: Rhinovirus Attenuates Non-typeable Hemophilus influenzae-stimulated IL-8 Responses via TLR2-dependent Degradation of IRAK-1
Source: PLoS Pathog. 2012 Oct 4;8(10):e1002969. doi: 10.1371/journal.ppat.1002969 (PMC3464227; doi:10.1371/journal.ppat.1002969)

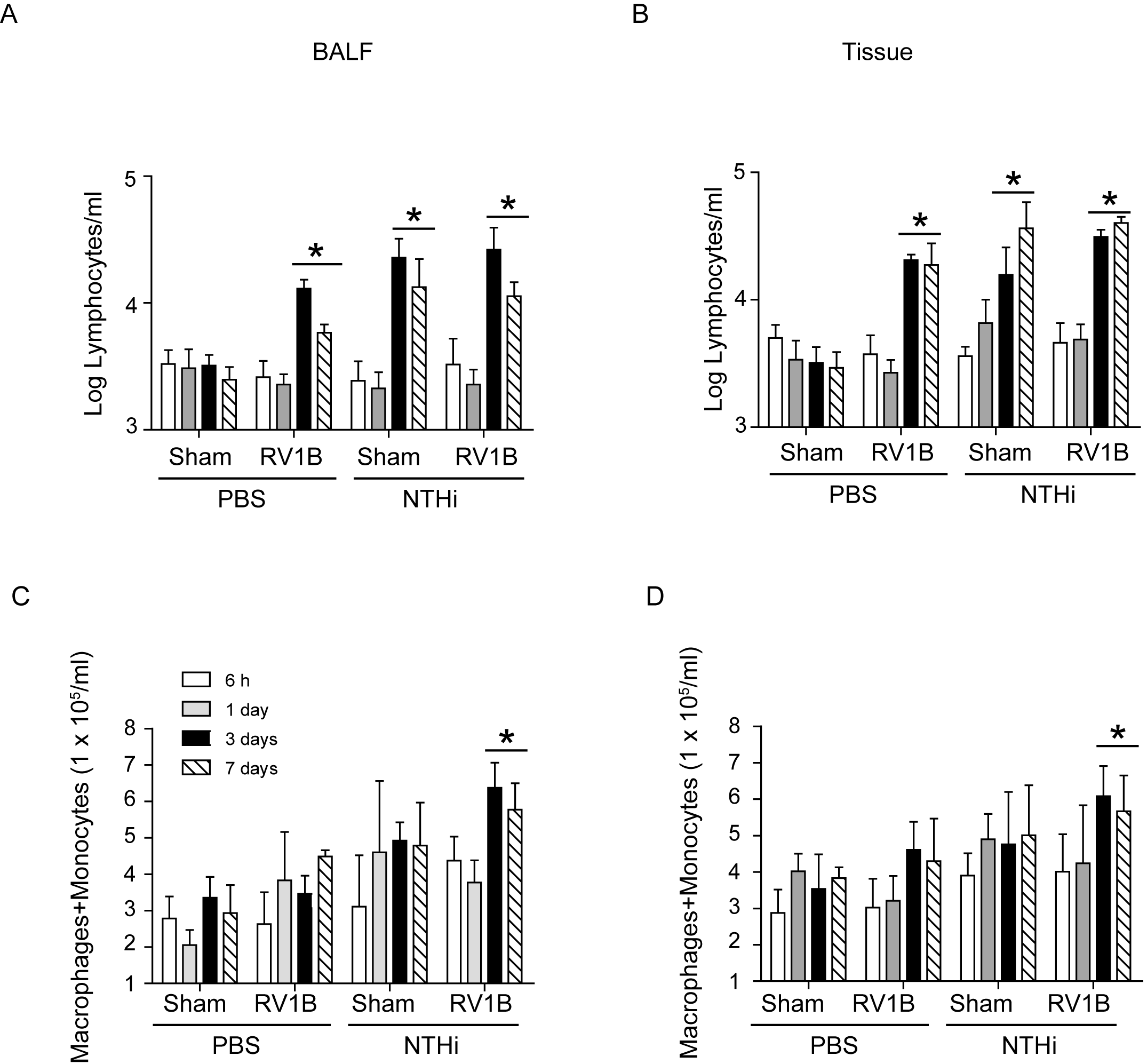

Supplement: Figure S1 — RV/NTHi infection does not alter macrophage/monocyte and lymphocytes in mice. BALB/C mice were infected with RV1B or sham by intranasal route. Two days later, mice were infected with NTHi or treated with PBS via intratracheal route and sacrificed at 6 h, 1 day, 3 days or 7 days post-NTHi infection. Cytospins of BAL cells and leukocyte enriched fraction from lung homogenates were prepared, stained with Diffquick and number of (A and B) lymphocytes and (C and D) macrophage/monocytes were counted. Data represent mean±SD (n = 5, *p≤0.05, two-way ANOVA, different from sham-infected animals). (TIF) [file ppat.1002969.s001.tif]

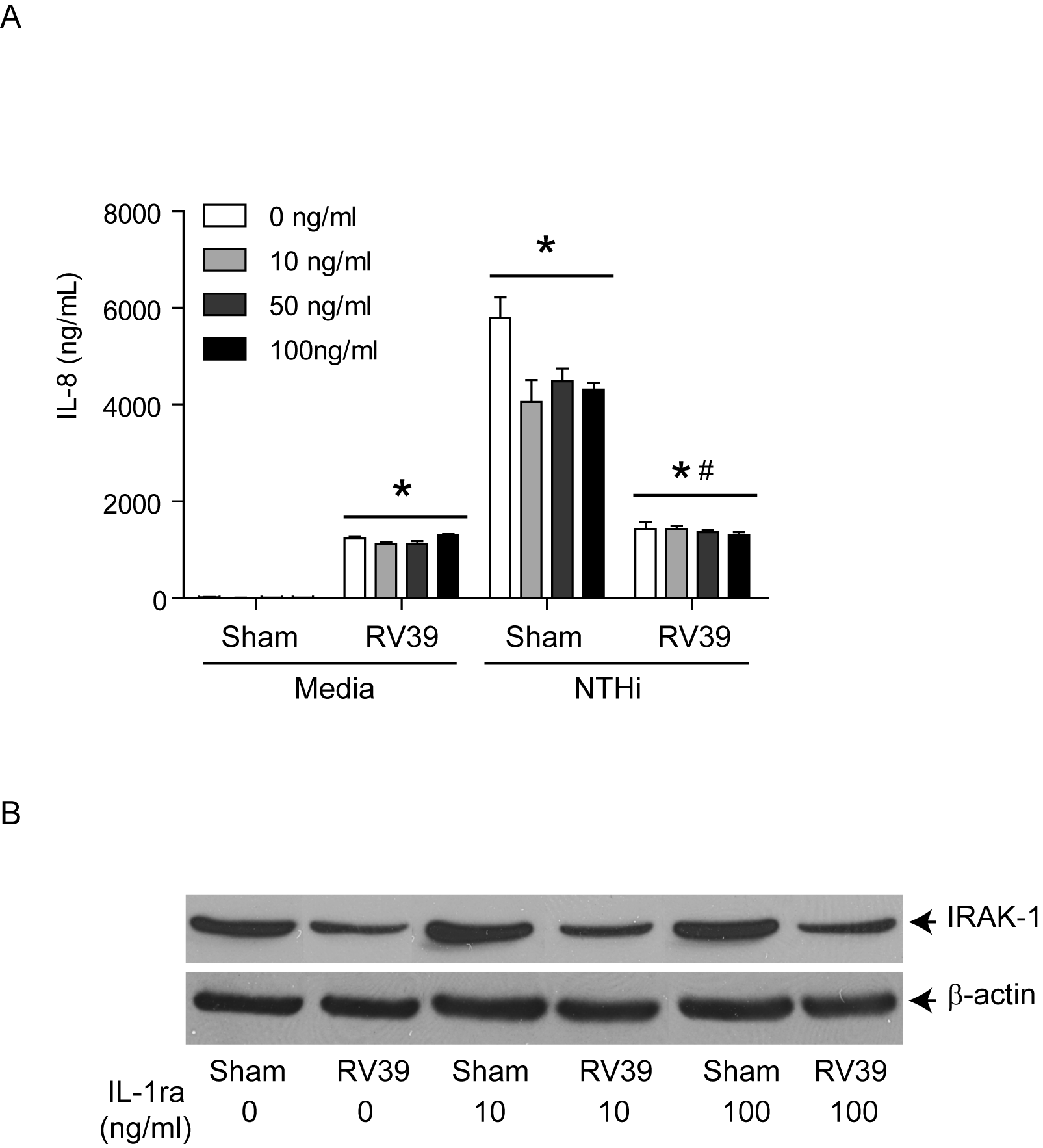

Supplement: Figure S2 — RV induced reduction in IRAK-1 protein is not IL-1β-dependent. BEAS-2B cells were infected with sham or RV39 and incubated at 33°C for 90 min. Infection media was replaced with media or media containing IL-1ra and incubation continued for another 22 h. (A) Cells were washed once with fresh media and then infected with NTHi and IL-8 in the media was assessed after 3 h. Data represents mean±SEM calculated from 3 independent experiments performed in triplicates (* p≤0.05, ANOVA, different from sham; #p≤0.05, ANOVA, different from sham/NTHi). (B) Cells were lysed following RV infection and the lysates corresponding to equal amounts of protein was subjected to Western blot analysis with IRAK-1 antibody. Image is a representative example of 2 independent experiments. (TIF) [file ppat.1002969.s002.tif]

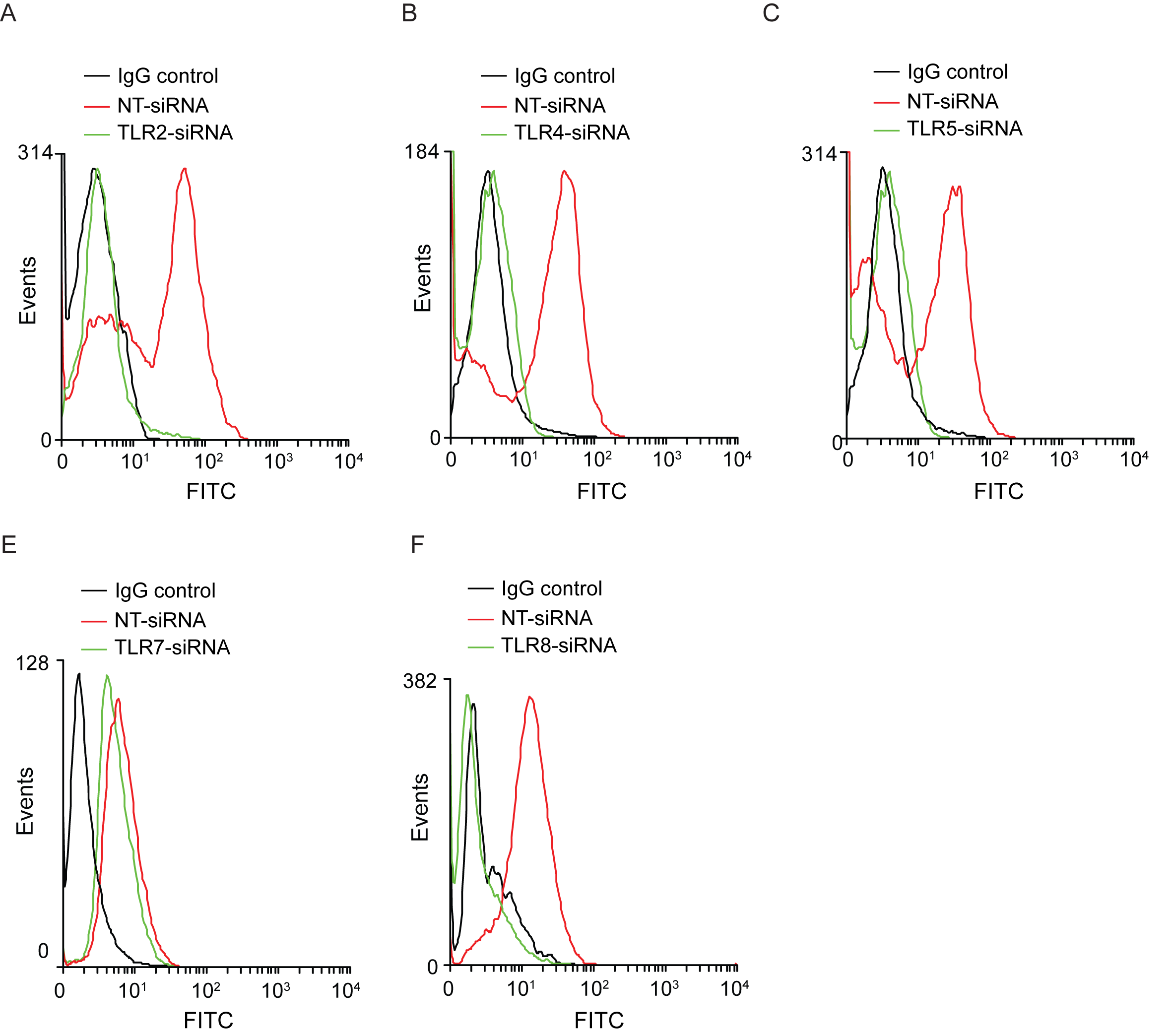

Supplement: Figure S3 — Gene specific siRNA to TLRs inhibits protein expression of respective TLRs. BEAS-2B cells were reverse transfected with NT-, TLR2, TLR4, TLR5, TLR7 or TLR8. After 48 h incubation, cells were either permeabilized (for TLR7 and TLR8) or not (TLR2, TLR4 and TLR5), incubated with antibodies to (A) TLR2, (B) TLR4, (C) TLR5, (D) TLR7 or (E) TLR8 or normal IgG conjugated with FITC and analyzed by flow cytometry. Image is a representative example of 2 independent experiments. (TIF) [file ppat.1002969.s003.tif]

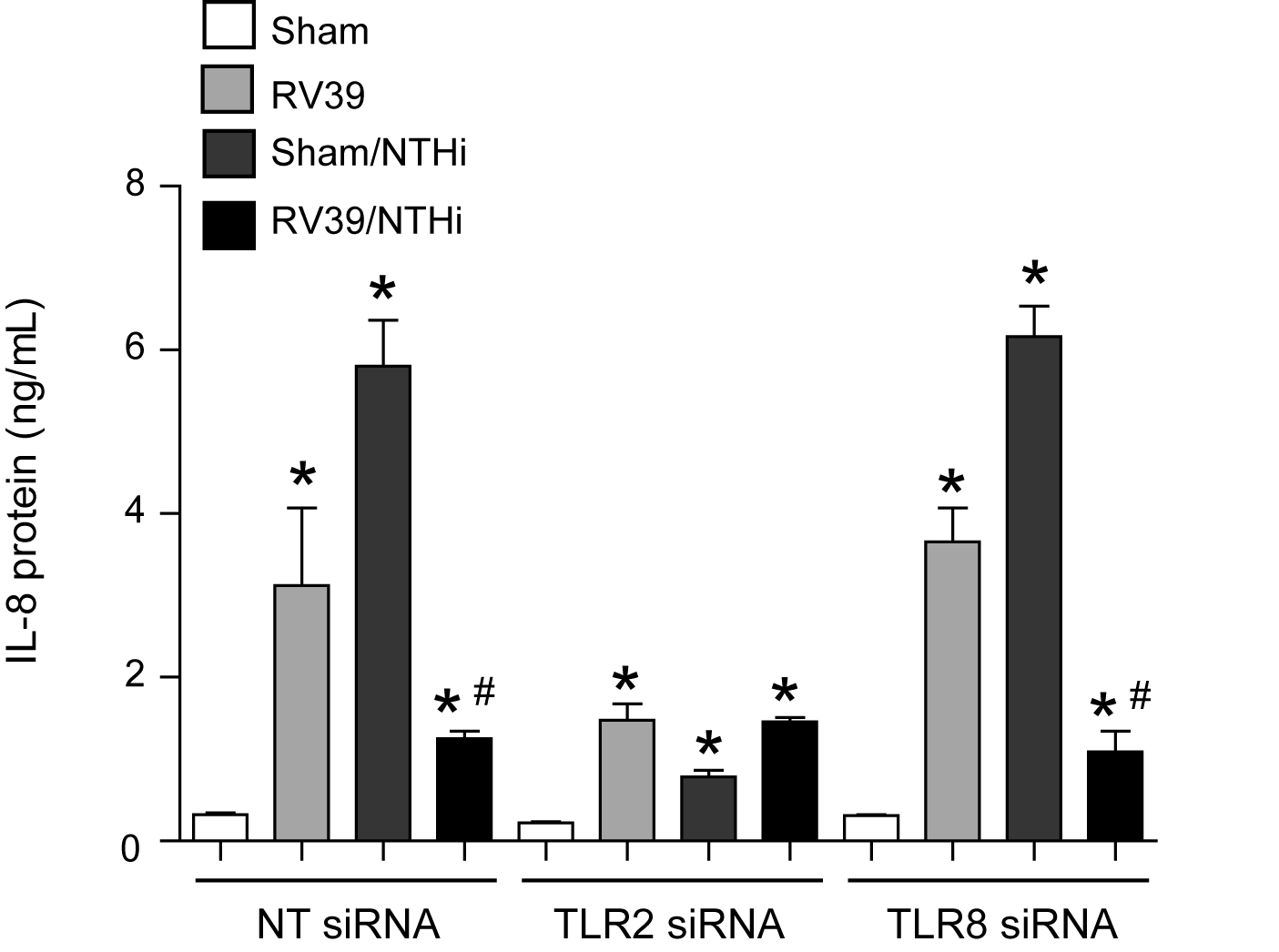

Supplement: Figure S4 — Knockdown of TLR2 decreases NTHi-stimulated IL-8. BEAS-2B cells reverse transfected with NT- or TLR2 siRNA were grown for 2 days. Cells were infected with NTHi or sham infected with media, incubated for 3 h at 37°C and IL-8 the medium was determined. Data represents mean±SEM calculated from 3 independent experiments performed in triplicates (* p≤0.05, ANOVA, different from media control; #p≤0.05, ANOVA, different from cells transfected with NT siRNA and then infected with NTHi). (TIF) [file ppat.1002969.s004.tif]
